# Dual origins of the intracellular circadian calcium rhythm in the suprachiasmatic nucleus

**DOI:** 10.1038/srep41733

**Published:** 2017-02-03

**Authors:** Ryosuke Enoki, Daisuke Ono, Shigeru Kuroda, Sato Honma, Ken-ichi Honma

**Affiliations:** 1 Photonic Bioimaging Section, Research Center for Cooperative Projects, Hokkaido University Graduate School of Medicine, N15 W7, Kita-ku, Sapporo, 060-8638, Japan; 2 Department of Chronomedicine, Hokkaido University Graduate School of Medicine, N15 W7, Kita-ku, Sapporo, 060-8638, Japan; 3 Precursory Research for Embryonic Science and Technology (PRESTO), Japan Science and Technology Agency (JST), Saitama 332-0012, Japan; 4 Mathematical and Physical Ethology Laboratory, Research Institute for Electrical Science, Hokkaido University, N20 W10, Kita-ku, Sapporo, Japan

## Abstract

In mammals, the master circadian clock is located in the suprachiasmatic nucleus (SCN), where most neurons show circadian rhythms of intracellular Ca^2+^ levels. However, the origin of these Ca^2+^ rhythms remains largely unknown. In this study, we successfully monitored the intracellular circadian Ca^2+^ rhythms together with the circadian PER2 and firing rhythms in a single SCN slice *ex vivo*, which enabled us to explore the origins. The phase relation between the circadian PER2 and Ca^2+^ rhythms, but not between the circadian PER2 and firing rhythms, was significantly altered in *Cry1*/*Cry2* double knockout mice, which display a loss of intercellular synchronization in the SCN. In addition, in *Cry1*/*Cry2* double knockout mice, circadian Ca^2+^ rhythms were abolished in the dorsolateral SCN, but were maintained in the majority of the ventromedial SCN. These findings indicate that intracellular circadian Ca^2+^ rhythms are composed of an exogenous and endogenous component involving PER2 expression.

In mammals, temporal orders of physiology and behavior are controlled by the master circadian clock located in the hypothalamic suprachiasmatic nucleus (SCN)^1^. The SCN consists of approximately 20,000 neurons, each of which shows self-sustained circadian oscillations^2^,^3^. It is widely accepted that the cellular circadian rhythm is generated by an autoregulatory transcriptional and translational feedback loop (core loop) consisting of the clock genes *Period (Per) 1, Per*2, *Cryptochrome (Cry) 1, Cry*2, *Bmal1,* and *Clock*, and their protein products^4^. In the SCN, more than one regional oscillator has been proposed in which a group of oscillating neurons synchronized to each other behave differently from other regional oscillators^5^. The best example of this has been observed between oscillating groups of the dorsal SCN, where arginine-vasopressin (AVP)-positive neurons are abundant, and those in the ventral SCN, where vasoactive intestinal peptide (VIP)-positive neurons predominate^5^,^6^,^7^,^8^.

Calcium ions (Ca^2+^) are important for intracellular signaling. Once Ca^2+^ enters the cytosol, either from the extracellular space or from the intracellular Ca^2+^ stores, it exerts a variety of effects on proteins and regulates various cell functions. In the SCN, the circadian rhythms of intracellular Ca^2+^ (Ca^2+^ rhythms) have been reported at the level of individual neurons^9^ and the cellular network^10^,^11^. Shut-down of the input pathway by tetrodotoxin (TTX) reduces the amplitude of Ca^2+^ rhythms by approximately 30%, hypothesizing that intracellular Ca^2+^ levels are controlled by both input pathways to the core loop and output pathways from the loop to various cellular functions^10^. Thus, the inter-relationships between physiological output functions, intracellular Ca^2+^, and transcriptional and translational regulation of the clock genes require further understanding^12^,^13^.

To this end, we established an *ex vivo* method to simultaneously and continuously monitor intracellular Ca^2+^ level, clock gene expression, and spontaneous firing in the same region of a single SCN slice. By this method, we purposed to determine the origin(s) of major signal other than the core molecular feedback loop, which affects the phases of intracellular circadian rhythms in these measures. Instead of TTX to block the input pathway to the circadian Ca^2+^ rhythm, we used the SCN of CRY1/2 double knockout (*Cry1,2*^−/−^) mice, in which the cellular synchronization is substantially attenuated^14^,^15^,^16^. *Cry1/2*^−/−^ mice show significant circadian rhythms in clock gene expression in the SCN up to at latest postnatal day 14 but afterwards they lose the circadian rhythm because of a dysfunction of the neural network^14^,^15^.

In this study, we report that the phase relationship between circadian PER2 and Ca^2+^ rhythms, but not that between circadian PER2 and firing rhythms, was significantly altered in the *Cry1,2*^−/−^ SCN. We also found that the circadian Ca^2+^ rhythms were abolished in the dorsolateral region of the SCN; however, the rhythm was maintained in the ventromedial region of the SCN in *Cry1,2*^−/−^ mice. These findings indicate that intracellular circadian Ca^2+^ rhythms are determined by at least two factors: the endogenous component from the core loop and the exogenous component from the SCN neural network.

## Results

### Simultaneous recordings of PER2, intracellular Ca^2+^, and spontaneous firing in the SCN

To analyze the functional links between the circadian rhythms of Ca^2+^, PER2 expression, and spontaneous firing in the SCN, we combined three established methods for measuring each circadian rhythm^10^,^14^. The intracellular Ca^2+^ level was monitored by expressing genetically encoded Ca^2+^ sensors, GCaMP6s^17^, which were transfected into the SCN slice using adeno-associated virus (AAV)^10^,^18^. PER2 expression was measured using a bioluminescence reporter, and spontaneous firing was recorded by a multielectrode array dish (MED) with 8 × 8 electrodes of 20 × 20 μm.

**Figure 1:**
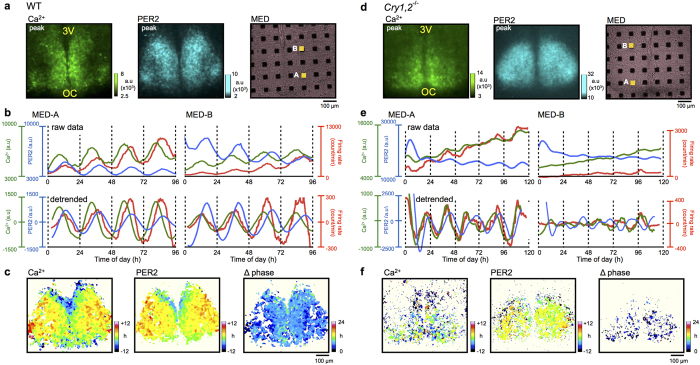
Simultaneous recordings of PER2, intracellular Ca^2+^, and spontaneous firing in the wild-type and *Cry1,2*^−/−^ SCN. Wild-type (**a–c**) and *Cry1,2*^−/−^ SCN (**d–f**) are displayed and compared. (**a,d**) Expression patterns of GCaMP6s fluorescence (left) and PER2::LUC bioluminescence (middle). 3 V, third ventricle; OC, optic chiasm. The SCN slice was cultured on a MED probe with 8 × 8 electrodes to measure spontaneous firing (right). (**b,e**) Two representative signals recorded on ROIs on MED electrodes. The Ca^2+^ rhythm (green), PER2 rhythm (blue), and spontaneous firing frequency rhythm (red) are displayed. Raw (top) and detrended data (bottom) are shown. Detrended data were calculated by subtracting the 24 h running average. (**c,f**) Acrophase maps of Ca^2+^ (left) and PER2 rhythms (middle), and the phase difference (Δ phase) maps (right). Peak phase time is depicted and normalized relative to the mean phase of the whole slice. Wild-type (WT): n = 70 ROIs in four slices, *Cry1,2*^−/−^. n = 47 ROIs in four slices. cP days of the SCN was 11–14 in WT and 11–15 *Cry1,2*^−/−^.

The SCN slice of PER2::LUC knock-in newborn mice was explanted on a culture membrane and AAV was transfected (Fig. S1a). Several days later, a flipped slice was placed on MED. Simultaneous recording of the three measures was started on the 10^th^ culture day, which corresponded to postnatal day 10. Circadian rhythms in GCaMP6s fluorescence (Ca^2+^ rhythms) and PER2::LUC bioluminescence (PER2 rhythms) were monitored by a high-sensitivity CCD camera mounted on an upright microscope, and spontaneous firing was monitored using a MED (Fig. S1b). Circadian Ca^2+^, PER2, and firing rhythms were analyzed in a region of interest (ROI) (20 × 20 μm) on each MED electrode (Fig. 1a).

**Figure 2:**
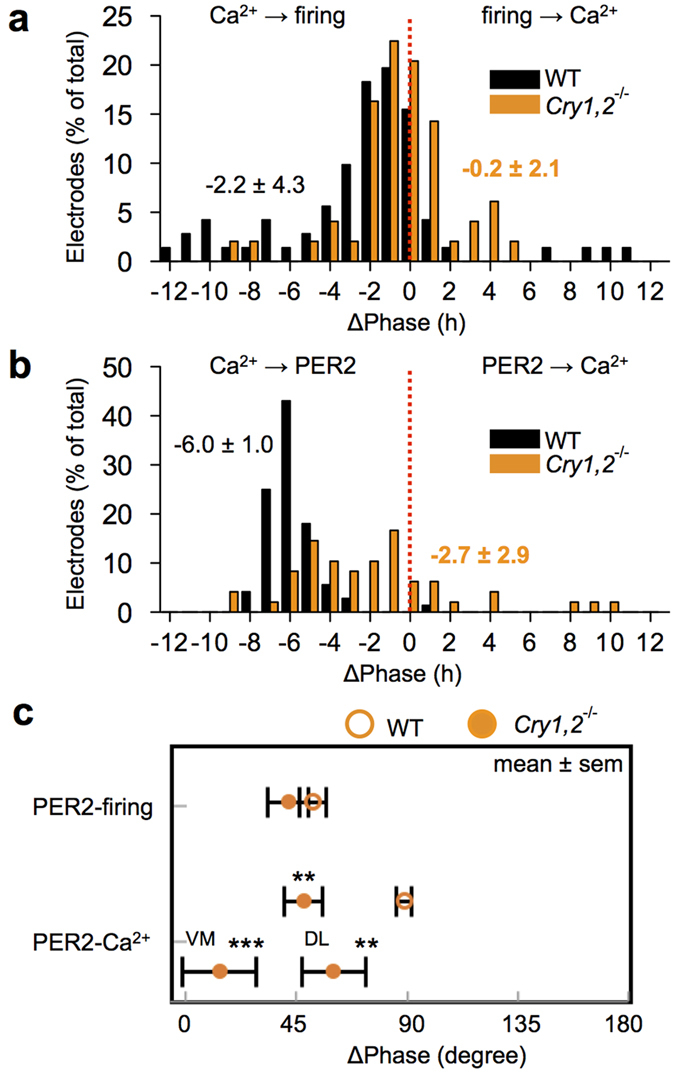
Statistical comparison of the phase differences in wild-type and *Cry1,2*^−/−^ SCN. (**a**) The phase difference (Δ phase) distribution between the Ca^2+^ and firing rhythm. (**b**) The Δ phase between the Ca^2+^ and PER2 rhythm. (**c**) Summary of the Δ phase (expressed as degree) relative to the PER2 rhythm in wild-type and *Cry1,2*^−/−^ SCN. Note that the mean Δ phase between PER2 and Ca^2+^ rhythms, but not between PER2 and firing rhythms, is smaller in *Cry1,2*^−/−^ than in wild-type SCN. **P < 0.01, ***P < 0.001. Wild-type (WT): n = 70 ROIs in four slices, *Cry1,2*^−/−^. n = 47 ROIs in four slices. ventromedial (VM): n = 25 ROIs, dorsolateral (DL): n = 22 ROIs. Degrees in WT and *Cry1,2*^−/−^ are equal to 4.2 h and 3.5 h, respectively.

In the wild-type SCN, robust circadian rhythms of Ca^2+^, PER2, and firing were detected in all ROIs examined in single SCN slices (Fig. 1b). The circadian peak of the Ca^2+^ rhythm was phase-advanced, relative to that of the PER2 rhythm, in the entire area of the SCN (Fig. 1c), showing a phase difference of approximately 6 h on average (Fig. 2b). The circadian peak of the firing rhythm was located between that of the Ca^2+^ and PER2 rhythms, and was phase-advanced to the circadian PER2 rhythm by 2.2 h on average (Figs 1b and 2a). In terms of SD, the phase relation between the circadian Ca^2+^ and firing rhythms was unstable compared with that between the Ca^2+^ and PER2 rhythms [4.3 vs. 1.0, p < 0.001 (Fig. 2a,b)]. The circadian Ca^2+^ and firing rhythms were occasionally (approximately 10% of total electrodes) 180 degrees out of phase (Fig. 2a).

### Changes in the circadian organization in the *Cry*1/2^−/−^ SCN

The *Cry1,2*^−/−^ SCN exhibited circadian rhythms for all three functions, but they were less robust and more variable compared to those of the wild-type (Fig. 1d–f). The circadian periods of all rhythms were significantly shorter and distributed in a wider range in the *Cry1,2*^−/−^ SCN than in the wild-type (Fig. S2). Two peaks appeared to exist in the period distributions, with one located at approximately 23 h, similar to that of the wild-type rhythm, and the other, substantially shorter, at approximately 18 h (Fig. S2). Circadian periods were significantly different between wild-type and *Cry1,2*^−/−^ SCN [Ca^2+^ : 24.4 ± 1.0 h vs. 20.0 ± 3.2 h, PER2: 24.9 ± 0.8 h vs. 20.6 ± 3.2 h, Firing: 24.1 ± 1.5 h vs. 20.0 ± 3.1 h (70 ROIs in 4 wild-type slices, 52 ROIs in 4 *Cry1,2*^−/−^ slices)]. Therefore, circadian phases of the *Cry1,2*^−/−^ SCN were described in terms of degrees [1 degree = 360 degree/period (Fig. 2c)]. The phase relation between the Ca^2+^ and PER2 rhythms was changed in the *Cry1,2*^−/−^ SCN [47.2 ± 7.8 degrees, n = 45 (Fig. 2c)], showing a significantly smaller phase difference than in the wild-type (87.8 ± 3.0 degrees, n = 72). Similarly, the phase difference between the Ca^2+^ and firing rhythms was smaller in the *Cry1,2*^−/−^ SCN (6.1 ± 11.5 degrees, n = 47) than in the wild-type (37.1 ± 8.0 degrees, n = 71). However, the phase relation between the PER2 and firing rhythms was not changed in the *Cry1,2*^−/−^ SCN (41.1 ± 8.3 degrees, n = 47) compared to that in the wild-type (50.7 ± 5.5 degrees, n = 71) (Fig. 2c). When the phase difference between the circadian Ca^2+^ and PER2 rhythms was separately calculated in the ventromedial and dorsolateral regions of the SCN, the difference was significantly larger in the dorsolateral (59.3 ± 12.9 degrees, n = 22) than ventromedial region [13.1 ± 14.9 degrees, n = 25 (Fig. 2c)]. These findings indicate that the circadian organization of the SCN, particularly in the ventromedial region, was significantly changed in the *Cry1,2*^−/−^ mice.

### Regional specificity of circadian Ca^2+^ and PER2 rhythms in the neonatal SCN

**Figure 3:**
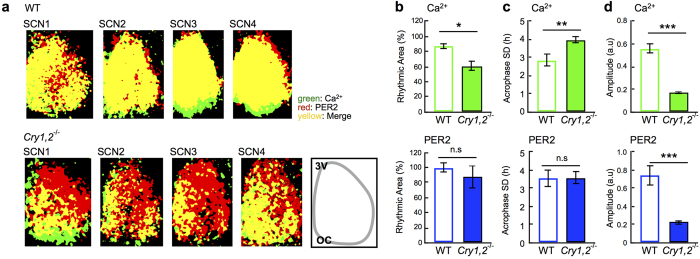
Statistical comparison of the network rhythmicity and coherence. (**a**) Rhythmic areas are compared by depicting pixels with significant Ca^2+^ rhythms in green, those with significant PER2 rhythms in red, and those with both rhythms in yellow. All SCN slices examined are shown for wild-type (upper) and *Cry1,2*^−/−^ SCN (lower). The percentage of the rhythmic areas in the SCN was estimated as the ratio of statistically rhythmic pixels (by Percent Rhythm, p < 0.01) (see Methods) to total pixels blighter than background level (>mean + 5 SD). (**b**) Percentage of the rhythmic area of Ca^2+^ (upper) and PER2 (lower) rhythms in wild-type and *Cry1,2*^−/−^ SCN. (**c**) The SD of acrophase for Ca^2+^ (upper) and PER2 rhythms (lower) in wild-type and *Cry1,2*^−/−^ SCN. (**d**) Amplitude of the Ca^2+^ (upper) and PER2 (lower) rhythms in wild-type and *Cry1,2*^−/−^ SCN. Amplitude and acrophase SD were obtained using pixel-based mapping software, *p < 0.05, **p < 0.01, ***p < 0.001, n.s (not significant). Wild-type (WT): n = 4 slices, *Cry1,2*^−/−^: n = 4 slices.

Circadian Ca^2+^ and PER2 rhythms in the wild-type SCN showed regional specificity in a coronal slice. For regional comparison of the circadian Ca^2+^ and PER2 rhythms, we used pixel-level analysis^10^,^19^,^20^. Note that each pixel has 2.3-μm resolution, which is smaller than the size of cell body. We found that the circadian peak phases of Ca^2+^ and PER2 rhythms were relatively phase-delayed in the narrow area from the dorsal to the medial border of the third ventricle and in the ventral area (Fig. 1c). The regional specificity of the circadian phases was not prominent in the *Cry1,2*^−/−^ SCN (Fig. 1f). Pixel-level analysis also revealed that a large area of the dorsolateral SCN in the *Cry1,2*^−/−^ mice was lacking circadian Ca^2+^ rhythms but did display circadian PER2 rhythms (Fig. 3a,b top). Whereas no difference was observed in the area with significant circadian PER2 rhythms (Fig. 3b bottom). The deviation in acrophase, an index of network synchronization, was significantly increased in the circadian Ca^2+^ rhythms of the *Cry1,2*^−/−^ SCN (Fig. 3c top), whereas there was no difference in the circadian PER2 rhythms (Fig. 3c bottom). On one hand, the amplitude was significantly reduced in the *Cry1,2*^−/−^ SCN for both circadian rhythms (Fig. 3d).

## Discussion

In the present study, we purposed to understand the functional links between circadian rhythms of three measures in the identical regions of same SCN tissue and successfully recorded the Ca^2+^, PER2, and firing rhythms simultaneously in single-cultured neonatal SCN slices. To our knowledge, this is the first study in which these rhythms have been continuously monitored together. The phase relationships between these circadian rhythms were substantially changed in the *Cry1,2*^−/−^ SCN. The circadian Ca^2+^ rhythm was not detected in the dorsolateral part of the *Cry1,2*^−/−^ SCN where AVP-containing neurons are predominantly located^10^. These findings indicate that the circadian Ca^2+^ rhythm is dissociable from the core loop and has dual origins: one of exogenous origin, the circadian input from the SCN network, and the other of endogenous origin, the intracellular circadian oscillation.

### Changes in the phase relationship between circadian PER2, Ca^2+^, and firing rhythms in the *Cry1,2* ^−/−^ SCN

Intracellular Ca^2+^ in the SCN has been demonstrated to oscillate in a circadian fashion^9^,^10^,^11^,^21^, and to show a stable phase relation with circadian firing^9^ and PER2 rhythm^10^,^11^. It has been reported that Ca^2+^ levels are mediated by intracellular stores^9^,^22^; blocking Ca^2+^ influx abolishes the rhythmic expression of clock genes in the SCN^23^. In these previous reports, the circadian peak of intracellular Ca^2+^ levels phase advanced to the circadian firing peak, indicating that the intracellular Ca^2+^ level was not a direct consequence of neuronal activity^9^. The present study confirmed these findings in wild-type mice by demonstrating a 2.2 h phase advance of the circadian Ca^2+^ rhythm to the firing rhythm. Importantly, the phase relation of the two rhythms was changed in the *Cry1,2*^−/−^ SCN and the phase difference was abolished (Fig. 2). Thus, the circadian Ca^2+^ rhythm and the firing rhythm in the SCN are dissociable. On the other hand, the phase relation between circadian Ca^2+^ and PER2 rhythms was also changed in the *Cry1,2*^−/−^ SCN. Interestingly, the phase relation was kept unchanged between the circadian PER2 and firing rhythms. These findings indicate that the circadian rhythm of intracellular Ca^2+^ in the wild-type SCN is regulated by at least two circadian oscillations of different origins, one generated by the core loop, represented by the circadian PER2 rhythm, and the other is the rhythmic input from the SCN neural network.

### Cellular mechanisms of circadian Ca^2+^ rhythms in the SCN

Ca^2+^ in the input pathways is critical for resetting the core loop and, thus, the coherence of the cellular circadian rhythms in the SCN network. Ca^2+^ modifies clock gene expression via the cAMP-responding element (CRE)^11^,^13^ and/or CaMKII activation, which activates E-box dependent gene expression through the phosphorylation of CLOCK and the dimerization with BMAL1^24^. In addition, Ca^2+^ is critical for the output pathways as it triggers release of transmitter and modulates action potentials via Ca^2+^ activated channels, including BK channels^25^. Blocking neuronal firing by TTX reduces the amplitude of circadian Ca^2+^ rhythms by only approximately 30%^10^. Therefore, the remaining circadian rhythm could be under the control of an endogenous oscillation(s). Thus, two factors of different origins may contribute to the circadian Ca^2+^ rhythm.

**Figure 4:**
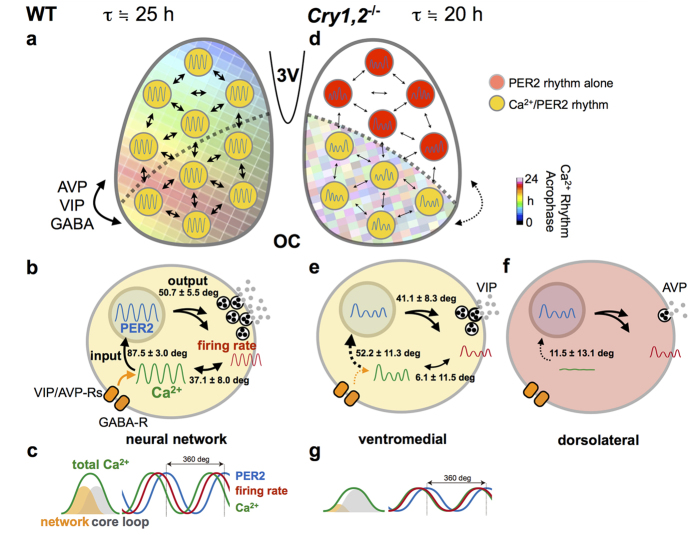
A schematic model of circadian organization in the SCN at the cellular and network levels. (**a~c**) In wild-type SCN (WT), the Ca^2+^ rhythms have unique topological patterns across the dorsal–ventral regions of SCN network, as indicated by the acrophase (**a**). SCN neurons communicate via neurotransmitters, such as GABA, VIP, and AVP. (**b**) At the cellular level, the Ca^2+^ rhythm is phase-advanced relative to the PER2 rhythm. The Ca^2+^ rhythms were also phase-advanced relative to the firing rhythms. Numbers in the cell indicates phase differences between the two parameters expressed in degrees. (**c**) Schema showing the order of the Ca^2+^ (green), firing (red), and PER2 (blue) rhythms (right). The phase and amplitude of Ca^2+^ rhythms are determined by at least two origins; the neuronal network (orange) and the core feedback loop (gray) (left). (**d~g**) In *Cry1,2*^−/−^ SCN, topological patterns of the Ca^2+^ rhythm are disorganized as indicated by the acrophase (**d**). In particular, the Ca^2+^ rhythms are almost undetectable in the dorsolateral region of the SCN where AVP-producing neurons are predominated. In the *Cry1,2*^−/−^ SCN, inter-cellular coupling is weaker than wild-type SCN. (**e**) At the cellular level, the amplitude of the rhythms was smaller, and the period was shorter than those in the wild-type SCN. Note that the mean Δ phase between the PER2 and Ca^2+^ rhythms became smaller in the *Cry1,2*^−/−^ SCN. Similarly, the phase difference between the Ca^2+^ and firing rhythms became smaller in the *Cry1,2*^−/−^ SCN. (**f**) The Ca^2+^, but not PER2, rhythm seldom oscillates in the dorsolateral region of the SCN, which results in altered release of transmitters, such as AVP^15^. (**g**) Schema showing the order of the three rhythms in *Cry1,2*^−/−^ SCN (right). The phase and amplitude of Ca^2+^ rhythms are primarily determined by the core loop (gray), while the contribution of the network (orange) is small (left). Degrees in WT and *Cry1,2*^−/−^ are equal to 4.2 h and 3.5 h, respectively.

*Bmal1* was reported to be critical for circadian Ca^2+^ rhythms. Suppression of *Bmal1* expression results in amplitude reduction and period elongation of Ca^2+^ rhythms in single SCN cells^26^. *Bmal1* expression is regulated by an auto-feedback loop^2^ which is interlocked with the core loop^2^,^3^, and is dissociable from the core loop represented by circadian PER2 rhythms^27^. The change in the phase relationship between circadian PER2 and Ca^2+^ rhythms could be interpreted as the result of the recoupling of two intracellular oscillations in the absence of circadian input from the SCN neural network. Alternatively, the two factors may contribute to the shape formation of circadian Ca^2+^ rhythms. The circadian Ca^2+^ rhythm consists of an endogenous component and an exogenous masking component from the SCN neural network. CRY double deficiency and TTX may block the latter component and deform the circadian Ca^2+^ rhythm, which apparently changes the phase relationship between them (Fig. 4).

### Regional specificity of circadian Ca^2+^ rhythms in the SCN

The change in the phase relationship among three cellular circadian rhythms in the *Cry1,2*^−/−^ SCN could be explained by regional specificity of the circadian network in the SCN. Circadian Ca^2+^ rhythms on the cellular level were ubiquitous in the wild-type SCN with a phase advance in the dorsal region of the SCN. Previously, TTX treatment in cultured SCN slices was shown to dissociate the circadian Ca^2+^ rhythms in the dorsal and ventral regions of the SCN^10^. In this case, the circadian Ca^2+^ rhythms in the ventral region were phase-delayed, showing a larger phase difference between the two regions. If the phase delay shifts by TTX were specific to the circadian Ca^2+^ rhythms, the phase relation with other circadian rhythms would also be changed.

On the other hand, the *Cry1,2*^−/−^ SCN lacks the circadian Ca^2+^ rhythm in the dorsolateral region, despite the expression of circadian PER2 rhythms (Figs 1f and 3a), although the amplitude was much smaller than wild-type (Fig. 3d). Since this region corresponds to the area of AVP-containing neurons, the Ca^2+^ rhythms may be critical for proper functioning of transmitter release. Recently, dysfunction of AVP signaling was demonstrated to be responsible for the loss of coherent circadian rhythms in the *Cry1,2*^−/−^ SCN^15^,^28^. An abolishment of circadian rhythms by AVP neuron-specific Bmal1 knockout in this region is known to deteriorate the behavior rhythms by splitting the activity onset and offset^7^. These findings indicate that AVP neurons in the dorsal regions of the *Cry1,2*^−/−^ SCN lose their mutual couplings for coherent circadian rhythms, as well as their communication to the ventral region of the SCN. We recently reported that AVP content is extremely reduced in *Cry1,2*^−/−^ SCN both in neonates and adults, and found that AVP and VIP signaling is differentially involved in the network of cellular oscillations depending on the developmental stages^15^. A lack of circadian signals from the dorsal to ventral region may change the phase relation between circadian PER2 and Ca^2+^ rhythms in these neurons.

In conclusion, the *Cry1,2*^−/−^ SCN shows an altered phase relation of circadian Ca^2+^ and PER2 rhythms in the ventral region, where the neural communication from AVP-containing neurons in the dorsal regions was substantially attenuated. The altered phase relation in the *Cry1,2*^−/−^ SCN was well explained in terms of dual signals which contribute the phase and/or the shape of intracellular Ca^2+^ rhythms and have different origins: a signal from the core molecular feedback loop and a signal from the neural network in which the cell is involved.

## Methods

### Animal ethics

All experimental protocols and methods performed were approved by the animal experiment ethics committee at Hokkaido University (approval no. 15–0153), and were in accordance with Hokkaido Univesity guidelines for the care and use of laboratory animals.

### Animal care

C57BL/6 J background mice were used for control experiments. *Cry1,2*^−/−^ mice, originally obtained from Tohoku University, were backcrossed with C57BL/6 J mice for more than 13 generations in our animal facility, and were bred with PER2::LUC mice carrying a PER2 fusion luciferase reporter. Mice were born and bred in our animal quarters under controlled environmental conditions (temperature: 22 ± 2 °C, humidity: 60 ± 5%, 12 h light/12 h dark, with lights on from 0600 to 1800 h). Light intensity was around 100 lx at the cage surface. The mice were fed commercial chow and tap water ad libitum.

### SCN slice culture

Decapitation was performed at the middle of the light phase. The brains of neonate mice (1-day old, both male and female) were rapidly removed and dipped in ice-cold balanced salt solution comprising (in mM) 87 NaCl, 2.5 KCl, 7 MgCl_2_, 0.5 CaCl_2_, 1.25 NaH_2_PO_4_, 25 NaHCO_3_, 25 glucose, 10 HEPES, and 75 sucrose. A 200 μm coronal brain slice containing the mid rostrocaudal region of the SCN was carefully prepared using a vibratome (VT 1200; Leica). We confirmed previously that the dorsolateral and ventromedial regions mostly contained AVP and VIP neurons, respectively^10^. The bilateral SCNs were dissected from the slice using a surgical knife and explanted onto a culture membrane (Millicell CM; pore size, 0.4 μm; Millipore) in a 35-mm Petri dish containing 1.0 mL of DMEM (Invitrogen) and 5% FBS (Sigma-Aldrich). Before the recordings, membranes with cultured SCN slices were cut out, flipped over, and transferred to glass-base dishes (35 mm, No.1-S; AGC Techno Glass) or MED dishes (MED-P210A) that were collagen coated (Cellmatrix type 1-c; Nitta Gelatin) and supplemented with 180–250 μL DMEM and 5% supplement. MED dishes were sealed with O_2_-permeable filters (membrane kit, High Sens; YSI) using silicone grease compounds (SH111; Dow Corning Toray).

### Adeno-associated virus-mediated gene transfer into SCN Slices

Aliquots of the adeno-associated virus (AAV, serotype 2/1) (1 μL) harboring GCaMP6s under the control of the human synapsin-1 promoter (produced by the University of Pennsylvania Gene Therapy Program Vector Core: https://www.med.upenn.edu/gtp/vectorcore/) were inoculated onto the surface of the SCN cultures 1 d after the preparation of the SCN slices. Infected slices were further cultured for at least 8 d before imaging. The titer of the GCaMP6s vector was 1.89 × 10^13^ genome copies/mL. We validated the specificity and transduction efficiency of AAV encoding hSyn-driven calcium indicator^10^. The calcium probes were expressed in SCN neurons, but not in glial cells, in the entire regions including the dorsolateral and ventromedial SCN, and the transduction efficiency was nearly 100%.

### Simultaneous recording of fluorescence, bioluminescence, and spontaneous firing

The SCN slice was cultured in 100% air on a MED dish with 8 × 8 electrodes of 20 × 20 μm. Before the recording, the MED dish was placed in a mini-incubator installed on the stage of the microscope (ECRIPSE 80i, Nikon). GCaMP6s fluorescence, PER2::LUC bioluminescence, and spontaneous firing were simultaneously recorded while culturing with the culture media supplemented with 0.5 mM D-luciferin K. Fluorescence and bioluminescence were recorded with a cooled CCD camera at −80 °C (512 × 512 pixels, 2.3 × 2.3 μm in pixel size) (ImagEM, Hamamatsu Photonics) every 60 min with an exposure time of 2–3 s for fluorescence and 3540 s (59 min) for bioluminescence, respectively, for 3–6 days. As exposure time was triple digits different, the spectrum overlap was negligible. GCaMP6s was excited at cyan color (475/28 nm) with an LED light source (Light Engine; Lumencor Inc) and the fluorescence was visualized with a 495-nm dichroic mirror and 520/35-nm emission filters (Semlock). Fluorescence and bioluminescence signals were analyzed in a region of interest (ROI) on each MED electrode (20 × 20 μm). The signal intensity was expressed as the average intensity of pixels involved in a ROI. Multi-unit spontaneous firings were recorded using an MED 64 system (Alpha MED Scientific). Spike discharges with signal–noise ratio >2.0 were collected by Spike Detector software (Alpha MED Scientific) as previously described^14^. The number of spikes per min was calculated for each electrode. Peak phases of the rhythms were estimated by the midpoint of rising and falling limbs of detrended circadian rhythms that intersected the x-axis.

### Data analysis and statistics

Statistical analyses were performed using Prism GraphPad (GraphPad Software). The group mean is presented as the mean ± sem. The student’s t-test was used when two independent group means were compared, and Welch’s t-test was used when the variances of two group means were different. Ventromedial and dorsolateral regions were selected based on the rhythmic area in Fig. 3.

For quantification of the rhythms in the SCN network, we used a custom-made program for creation of acrophase maps, as described previously^7^,^10^,^19^,^20^. Briefly, fluorescence and bioluminescence images were smoothed with the median filter (one pixel), and converted to eight-bit intensity. The time series of the images in each pixel, {Yj (ti); ti = 1,2, …, N (h)}, was fitted to a cosine curve, with yj(t) = yj (t; Mj, Aj, Cj, Tj) = Mj + Aj * cosine (2pi (t − Cj)/Tj), using a least-squares regression method, where j, yj (t) is the signal intensity at time t (h), Mj is the mesor, Aj is the amplitude, Cj is the acrophase, and Tj is the period of the images. The goodness of fit was statistically evaluated by Percent Rhythm accounted for by the fitted cosine wave (Pearson product–moment correlation analysis) at a significance level of p < 0.001. The percentage of the rhythmic areas in the SCN was estimated as the ratio of statistically rhythmic pixels (by Percent Rhythm, p < 0.01) to total pixels blighter than background level (>mean + 5 SD).

## Additional Information

**How to cite this article**: Enoki, R. *et al*. Dual origins of the intracellular circadian calcium rhythm in the suprachiasmatic nucleus. *Sci. Rep.* **7**, 41733; doi: 10.1038/srep41733 (2017).

**Publisher's note:** Springer Nature remains neutral with regard to jurisdictional claims in published maps and institutional affiliations.

## Supplementary Material

Supplemental Information
