# Supplementary material for: Dual origins of the intracellular circadian calcium rhythm in the suprachiasmatic nucleus
Source: Sci Rep. 2017 Feb 3;7:41733. doi: 10.1038/srep41733 (PMC5290527; doi:10.1038/srep41733)
Supplement: Supplemental Information [file srep41733-s1.pdf]

Supplemental Information

Dual origins of the intracellular circadian calcium rhythm in the suprachiasmatic nucleus

Ryosuke Enoki[1,2,3,*], Daisuke Ono[1,5,*], Shigeru Kuroda[4], Sato Honma[2], and Ken-ichi Honma[2]

Author affiliation:

[1] Photonic Bioimaging Section, Research Center for Cooperative Projects; [2] Department of Chronomedicine, Hokkaido University Graduate School of Medicine, N15 W7, Kita-ku, Sapporo 060-8638, Japan. [3] Precursory Research for Embryonic Science and Technology (PRESTO), Japan Science and Technology Agency (JST), Saitama 332-0012, Japan. [4] Mathematical and Physical Ethology Laboratory, Research Institute for Electrical Science, Hokkaido University, N20 W10, Kita-ku, Sapporo, Japan.

[5] Present address: Department of Neuroscience II, Research Institute of Environmental Medicine, Nagoya University, Nagoya 464-8601, Japan.

*These authors contributed equally to this work.

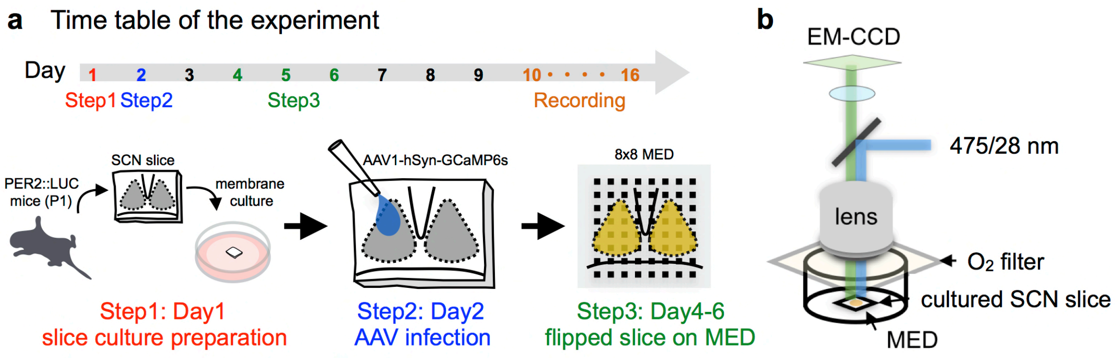

Fig. S1. Experimental procedure for simultaneous recording.
(a) Step 1 (Day 1): The SCN slice was prepared from PER2::LUC knock-in newborn mice of postnatal day 1, and was explanted on a culture membrane. Step 2 (Day 2): Aliquots of the adeno-associated virus (AAV) (1 µL) harboring GCaMP6s under the control of the human synapsin-1 promoter (hSyn) were inoculated onto the surface of the SCN cultures. Step 3 (Days 4–6): The membrane with the cultured SCN slice was cut out, flipped over, and transferred to a multielectrode array dish (MED) with 64 electrodes. Simultaneous recording of three measures was started from the 10th culture day. (b) The fluorescence of GCaMP6s and the bioluminescence of PER2 expression were measured by a high-sensitivity EM-CCD camera mounted on an upright microscope. Spontaneous firing was recorded by an MED system. The MED was sealed with an O_2-permeable filter. GCaMP6s was excited using 475/28 nm LED.

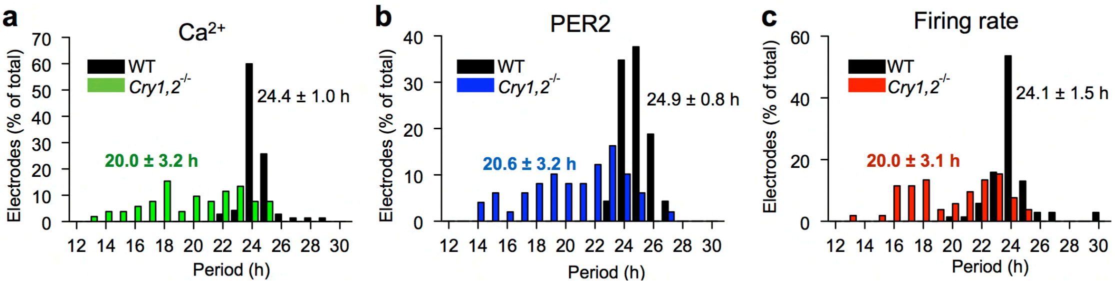

Fig. S2. Statistical comparison of the circadian period of Ca^{2+}, PER2, and spontaneous firing rhythms in the wild-type and *Cry1,2*$^{-/-}$ SCN.

The fluorescence/bioluminescence signals were obtained in ROIs on each MED electrode and the distributions of the circadian period were analyzed in wild-type and *Cry1,2*$^{-/-}$ SCN for Ca^{2+} (a), PER2 (b), and spontaneous firing rhythms (c). All data are given as means ± sd. Wild-type (WT): n = 70 in four slices, *Cry1,2*$^{-/-}$: n = 52 in four slices.
